# Health related quality of life and cardiac symptoms following admission for suspected MINOCA: A post-hoc analysis of the MINOCA-BAT randomized clinical trial

**DOI:** 10.1016/j.ahjo.2026.100856

**Published:** 2026-08-05

**Authors:** Bertil Lindahl, Anna Nordenskjöld, Sivabaskari Pasupathy, Rosanna Tavella, Stefan Agewall, Dan Atar, Tomasz Baron, Olle Bergström, David Erlinge, Chris P. Gale, Tomas Jernberg, Felicia Håkansson, Pelle Johansson, Javier López Pais, Annica Ravn-Fischer, Harmony Reynolds, Jithendra Somaratne, John Beltrame

**Affiliations:** a Department of Medical Sciences, Uppsala University, Uppsala, Sweden; b Department of Cardiology, Faculty of Medicine and Health, Örebro University, Örebro, Sweden; c School of Medicine, College of Health, Adelaide University, Australia; d Department of Clinical Sciences, Danderyd Hospital Division of Cardiovascular Medicine Karolinska Institutet Stockholm, Sweden; e Dept of Cardiology, Oslo University Hospital Ullevål, Oslo, Norway; f Department of Internal Medicine, Växjö Hospital, Växjö, Sweden; g Department of Cardiology, Clinical Sciences, Lund University, Skåne University Hospital, Lund, Sweden; h Leeds Institute of Cardiovascular and Metabolic Medicine, University of Leeds, Leeds, UK; i Department of Clinical Science and Education Södersjukhuset, Karolinska Institutet, Stockholm, Sweden; j The Swedish Heart and Lung Association, Stockholm, Sweden; k Department of Interventional Cardiology, Instituto Cardiovascular Barreiro y Asociados, Complexo Hospitalario Universitario de Santiago de Compostela, Spain; l Department of Molecular and Clinical Medicine, Institute of Medicine, Sahlgrenska Academy, University of Gothenburg, Sweden; m Center for Women's Cardiovascular Health, Cardiovascular Clinical Research Center, Leon H. Charney Division of Cardiology, Department of Medicine, NYU Grossman School of Medicine, United States of America; n Green Lane Cardiovascular Service, Auckland City Hospital, New Zealand; o Department of Cardiology, Central Adelaide Local Health Network, SA Health, Australia; p Institute of Clinical Medicine, University of Oslo, Norway; q Department of Cardiology, Leeds Teaching Hospitals NHS Trust, Leeds, UK; r Department of Cardiology, Sahlgrenska University Hospital, Gothenburg, Sweden

**Keywords:** Myocardial infarction (MI) with non-obstructive coronary arteries (MINOCA), Health related quality of life, Cardiac symptoms, Sex

## Abstract

**Background:**

Myocardial infarction (MI) with non-obstructive coronary arteries (MINOCA) accounts for 6–8% of all MIs and is more common in women than men. After an episode of MINOCA, patients may experience a substantial symptom burden and reduced health-related quality of life (HRQoL). The aim of this post-hoc analysis was to describe clinical characteristics, residual symptoms and HRQoL, including sex differences in patients with a working diagnosis of MINOCA and preserved left ventricular ejection fraction (LV-EF).

**Methods:**

Randomized Evaluation of **B**eta Blocker and **A**CE-Inhibitor/Angiotensin Receptor Blocker **T**reatment in MINOCA patients (MINOCA-BAT) was an international clinical trial. Patients with a working diagnosis of MINOCA and LV-EF ≥ 40% were randomized to beta-blocker versus no beta-blocker and ACEI/ARB versus no ACEI/ARB. The trial was prematurely terminated after 192 enrolled participants (median age 59; 63% women). Clinical events, symptoms and HRQoL (EQ-5D-3L) were assessed at 7 weeks and 12 months after the index event.

**Results:**

The cohort represented a low-risk population. At one year, one patient had died, one had a re-infarction, 14% reported chest pain and 9% dyspnea. Median EQ-5D index scores did not change significantly between 7 weeks and 12 months (0.86 vs. 0.78) and were comparable to age-matched general population norms. Women reported slightly lower HRQoL than men.

**Conclusions:**

Patients with a working diagnosis of MINOCA and preserved LV-EF constitute a low-risk population and demonstrate a favorable prognosis with symptom resolution within one year. Their HRQoL appears broadly similar to that observed in the general population, with only modest differences between the sexes.

## Highlights

• Patients with MINOCA generally report lower health-related quality of life (HRQoL).
• Post-hoc analysis of the MINOCA-BAT trial, which enrolled 192 patients with MINOCA and preserved left-ventricular ejection fraction (LV-EF), found
• A favourable prognosis and symptom resolution within one year
• HRQoL levels comparable to those of the general population
• A significant but small sex-related differences in HRQoL

## 1 Introduction

Approximately 6–8% of all patients with myocardial infarctions (MI) will receive a working diagnosis of suspected myocardial infarction with non-obstructive coronary arteries (MINOCA) [1]. Compared with patients who experience MI secondary to coronary artery disease (MI-CAD), those with MINOCA are generally younger, more frequently female and have fewer comorbidities [2], [3]. Although the risk of mortality and recurrent cardiovascular events after a MINOCA episode is lower than MI-CAD in general [2], it is comparable to that observed in patients with MI-CAD involving single- or two-vessel disease [4].

Prior studies suggest that the incidence of angina pectoris after a MINOCA episode is comparable to that observed following MI-CAD [5], and that MINOCA patients experience similar or lower Health Related Quality of Life (HRQoL) compared with MI-CAD patients after the index event [6]. However, no studies to date have described and compared HRQoL between men and women following a MINOCA episode. The importance of sex- and gender-related differences in risk factors, symptom presentation, and pathophysiology in myocardial infarction has recently been underscored in a scientific statement from the European Society of Cardiology [7].

Therefore, the aim of this post-hoc analysis of the MINOCA-BAT trial was to describe and compare clinical characteristics, as well as the prevalence of chest pain, dyspnea, and QoL at 1–2 months and 12 months between men and women with a working diagnosis of MINOCA and preserved left ventricular ejection fraction.

## 2 Methods

Randomized Evaluation of **B**eta Blocker and **A**CE-Inhibitor/Angiotensin Receptor Blocker **T**reatment in MINOCA patients (MINOCA-BAT) was a multicenter, prospective, randomized, controlled and open-label registry-based trial with 2:2 factorial design performed in Sweden, Australia, Spain, Norway and New Zealand [8]. The study was planned to recruit 3500 patients. The first patient was enrolled on January 23rd, 2019. The enrolment was prematurely terminated on September 14th, 2022, due to a slow recruitment rate. In total, 192 patients were recruited and randomized. Follow-up ended on May 31, 2023. The primary aim was to determine whether beta blockade compared to no beta blockade, and whether ACEI/ARB compared to no ACEI/ARB, reduce the composite primary endpoint in patients discharged following a clinical presentation with MINOCA but without clinical signs of heart failure and a left ventricular ejection fraction (LV-EF) ≥40%. The primary endpoint was a composite of time to death from any cause and readmission due to myocardial infarction, ischemic stroke or heart failure. The inclusion criteria were age ≥ 18 years, a clinical diagnosis of MINOCA within the last 30 days, LVEF ≥40% measured during hospitalization and prior to randomization, and written informed consent obtained. The exclusion criteria were any condition that may influence the patient's ability to comply with study protocol, previous revascularization, clinical signs of heart failure, cardiac magnetic resonance imaging proven myocarditis or a strong clinical suspicion of myocarditis as cause of the index event, contraindications for beta blocker or ACEI/ARB treatment, prior use of beta blocker, ACEI/ARB which must continue according to Investigator, new indication for beta blocker or ACEI/ARB treatment other than as secondary prevention according to Investigator, ongoing pregnancy or woman of childbearing potential not using highly effective contraceptives, participation in a trial evaluating a drug known to interact with beta blockers or ACEI/ARB. The patient had to meet the criteria for myocardial infarction according to the 4th Universal Definition of MI [9] and have no coronary diameter stenosis ≥50%. The degree of stenosis was visually assessed by the angiographer at each site, and no central adjudication of the coronary angiograms was performed. All patients were planned to have a physical visit/telephone contact at 7 weeks and one year and then followed either by mandatory registry data capture (Sweden) or regular visit/telephone contacts (non-Swedish Countries) for up to a mean of 4 years. At the visits after 7 weeks and 12 months the occurrence of angina (CCS class) or non-ischemic chest pain, and dyspnea (NYHA class) were reported. Patients also completed the EQ-5D (3 L) quality-of-life questionnaire, which covers five dimensions: mobility, self-care, usual activities, pain/discomfort, and anxiety/depression [10]. Each dimension has three levels of severity (no problems, some/moderate problems, or extreme problems). The EQ-5D index score was calculated using the European VAS value set [10].

The MINOCA-BAT trial was registered in ClinicalTrials.gov (NCT03686696) and approved by the appropriate Ethical Review Boards in each participating country, in accordance with the Declaration of Helsinki principles.

The occurrence of chest pain, dyspnea and of the EQ-5D index score were compared for the Swedish patients with individuals from the general population of the same age previously published [10] and with scores calculated in 26,645 patients of the same age with myocardial infarction (the vast majority type 1) and LV-EF ≥40% registered in the SWEDEHEART registry (approved by the Regional Ethical Review Board 2012/60-31/2).

### 2.1 Statistics

Continuous variables are presented as median and inter quartile range (IQR). Categorical variables are presented as frequencies. Comparisons between groups were performed using the Chi-square test, and paired comparisons were assessed with McNemar's test. For comparison of two unrelated samples on an ordinal or interval scale, the Mann-Whitney *U* test was used; and for comparison of two related samples the Wilcoxon signed-rank test was used. Linear regression models were constructed to predict the EQ-5D index score using baseline clinical variables. *P*-values ≤0.05 were considered significant. No adjustment of *p*-values for multiple comparisons was performed. IBM SPSS version 30.0 (IBM Corp., Armonk, NY) was used for statistical analyses.

## 3 Results

A total of 192 patients were randomized and followed for at least one year: 112 in Sweden, 67 in Australia, 5 in Spain, 5 in Norway, and 3 in New Zealand (the latter four countries are hereafter collectively referred to as non-Swedish). Forty-three patients were randomized to receive both beta-blockers and ACEI/ARB; 45 to neither beta-blockers nor ACEI/ARB; 55 to ACEI/ARB without beta-blockers; and 49 to beta-blockers without ACEI/ARB.

Clinical characteristics, admission details and laboratory results in the total study population and in men and women, are presented in Table 1. The study population had a median age of 59 years, a predominance of females, and relatively low rates of cardiovascular risk factors and comorbidities. Ninety-five percent were admitted with chest pain. On admission ECG, 96% had sinus rhythm, 13% showed ST-segment elevation, 11% ST depression but most (58%) had no ST-T changes. Invasive coronary angiography revealed no or only minimal atherosclerosis in 82% of patients, and 94% had LV-EF of 50% or higher. There were few significant differences in clinical characteristics, admission details, and laboratory results between men and women. Women were, on average, slightly older; fewer were prescribed P2Y12 inhibitors; their admission ECGs more frequently showed ST–T changes; they had lower hemoglobin and creatinine concentrations; were more likely to present with no or minimal coronary atherosclerosis; and were less likely to have a LV-EF of 50% or higher compared with men.

**Table 1:** Clinical characteristics, admission details and laboratory results.

	Total pop	missing	Men	Women	p-Value
	*N* = 192		*N* = 74	*N* = 118	
*Clinical characteristics*					
Age, median (25th–75th perc.)	**59** (**53**–**67**)	0	57 (51–64)	60 (54–69)	=0.04
Sex - women	**118** (**62%**)	0	–	–	
BMI, median (25th–75th perc.)	**27** (**24**–**31**)	7	28 (25–31)	27 (23−31)	NS
Occupation†					
Working	**60** (**56%**)		20 (61%)	40 (54%)	NS
Retired	**43** (**40%**)		13 (39%)	30 (40%)	
Other	**4** (**4%**)		0	4 (5%)	
Smoking					
Never	**87** (**46%**)	2	32 (44%)	55 (47%)	NS
Previous	**61** (**32%**)		21 (29%)	40 (34%)	
Ongoing	**42** (**22%**)		20 (27%)	22 (19%)	
Hypertension	**68** (**35%**)	0	26 (35%)	42 (36%)	NS
Diabetes	**14** (**7%**)	0	6 (8%)	8 (7%)	NS
Prev. MI	**9** (**5%**)	0	5 (7%)	4 (3%)	NS
CHF	**1** (**0.5%**)	1	0	1 (1%)	NS
Prev. stroke	**4** (**2%**)	0	2 (3%)	2 (1%)	NS

*Admissions details*					
Reason for admission		0			
Chest pain	**183** (**95%**)		**71** (**96%**)	**112** (**95%**)	=0.085
Dyspnea	**2** (**1%**)		**2** (**3%**)	**0**	
Other	**7** (**4%**)		**1** (**1%**)	**6** (**5%**)	
ECG					
Sinus rhythm	**164** (**96%**)	22	**62** (**98%**)	**102** (**95%**)	NS
Other	**6** (**4%**)		**1** (**2%**)	**5** (**5%**)	
ECG					
ST elevation	**18** (**13%**)	50	**6** (**12%**)	**12** (**13%**)	=0.005
ST-depression	**15** (**11%**)		**2** (**4%**)	**13** (**14%**)	
T-wave inv	**13** (**9%**)		**2** (**4%**)	**11** (**12%**)	
Other	**13** (**9%**)		**1** (**2%**)	**12** (**13%**)	
No ST-T change	**83** (**58%**)		**39** (**78%**)	**44** (**48%**)	
Heart rate	**78** (**66**–**89**)	2	76 (64–86)	80 (67–92)	NS
Systolic BP	**147** (**130**–**165**)	2	**145** (**130**–**160**)	**148** (**130**–**170**)	NS

*Medication at admission*					
ACEI	**16** (**8%**)	0	5 (7%)	11 (9%)	NS
ARB	**17** (**9%**)	0	5 (7%)	12 (10%)	NS
Beta-blocker	**20** (**10%**)	0	5 (7%)	15 (13%)	NS
Ca-inhibitor	**17** (**9%**)	0	5 (7%)	12 (10%)	NS
Long-acting nitrate	**0**	0	0	0	
Diuretics	**7** (**4%**)	0	3 (4%)	4 (3%)	NS
MRA	**2** (**1%**)	0	0	2 (2%)	NS
Statins	**40** (**21%**)	0	18 (24%)	22 (19%)	NS
Ezetimibe	**5** (**3%**)	0	3 (4%)	2 (2%)	NS
Diabetes treatment	**9** (**5%**)	0	4 (5%)	5 (4%)	NS
Aspirin⁎	**18** (**10%**)	18	9 (14%)	9 (8%)	NS
P2Y12-inh.	**18** (**9%**)	1	12 (16%)	6 (5%)	=0.009
Anticoagulant treatment	**11** (**6%**)	1	6 (8%)	5 (4%)	NS

*Laboratory results*					
Troponin T	**142** (**73**–**346**)	79	155(72–335)	127 (72–356)	NS
Troponin I	**450** (**115**–**1620**)	117	541 (122–1659)	440 (108–1525)	NS
CRP	**4** (**1.4**–**5**)	68	4 (1–5.7)	4 (2–5)	NS
Hb	**141** (**132**–**149**)	12	149 (139–155)	137 (128–144)	<0.001
Creatinine†	**68** (**60**–**82**)		80 (71–90)	64 (56–73)	<0.001
P-glucose†	**5.9** (**5.3**–**6.5**)		6.0 (5.4–6.3)	5.8 (5.3–7.2)	NS
Cholesterol	**4.9** (**4.4**–**5.8**)	38	4.6 (3.8–5.4)	4.9 (4.4–6.0)	=0.005
Coronary stenosis grade					
0–29%	**158** (**82%**)	0	54 (73%)	104 (88%)	=0.01
30–49%	**28** (**15%**)		18 (24%)	10 (8%)	
>50%	**6** (**3%**)		2 (3%)	4 (3%)	
Echocardiogr.					
LVEF ≥ 50%	**175** (**94%**)	5	71 (99%)	104 (90%)	=0.026
LVEF 40–49%	**12** (**6%**)		1 (1%)	11 (10%)	

† Only Swedish patients.

⁎ In 17 patients it was unclear whether Aspirin was started first after admission or the patient was treated with Aspirin before admission. Therefore, in these cases Aspirin treatment was set to missing.

Non-randomized medications prescribed at discharge are presented in Supplemental Table 1. Aspirin was prescribed to 88% of patients, statins to 79%, P2Y12 inhibitors to 47%, and calcium-channel inhibitors to 16%. Other medications were prescribed in fewer than 5% of cases. Statins and P2Y12 inhibitors were more frequently prescribed to men than to women; otherwise, no significant differences in treatment were observed between the sexes.

### 3.1 Follow-up

At the 7-week visit (median of 7 [interquartile range (IQR) 5–9] weeks after randomization), 5% of patients randomized to beta-blockers and 12% of those randomized to ACEI/ARB had discontinued treatment. Conversely, 6% and 11% of patients initially randomized to no beta-blockers and no ACEI/ARB, respectively, had crossed over and initiated treatment. At the 12-month visit (median 53 [IQR 51–55] weeks after randomization), the corresponding figures were 27%, 14%, 15%, and 16%. There were no significant differences in adherence between men and women (data not shown).

At 12 months very few primary endpoints had occurred: one non-cardiac death, one myocardial infarction, and no ischemic strokes or hospitalizations for heart failure.

### 3.2 Chest pain, dyspnea and Health Related Quality of Life at follow-up

At the 7-week visit after discharge, 80% of patients reported no chest pain, increasing to 86% at the 12-month visit. Correspondingly, 14% and 8% experienced mild angina (CCS class I–II), while 5% and 4% reported chest pain that were considered non-ischemic by the treating physician (Table 2). There was no difference in chest pain pattern between the sexes at the 7-week or 12-month visits.

**Table 2:** Chest pain and dyspnea at follow-up.

	Total pop N = 192	Missing	Men N = 74	Women N = 118	p-Value
*Chest pain*					
7-week visit		20			NS
No chest pain	**138** (**80%**)		55 (81%)	83 (80%)	
CCS I-II	**24** (**14%**)		11 (16%)	13 (13%)	
CCS III-IV	**2** (**1%**)		1 (2%)	1 (1%)	
Non-ischemic chest pain	**8** (**5%**)		1 (2%)	7 (7%)	
12-month visit		40			NS
No chest pain	**131** (**86%**)		52 (87%)	79(86%)	
CCS I-II	**12** (**8%**)		4 (7%)	8 (9%)	
CCS III-IV	**3** (**1%**)		2 (3%)	1 (1%)	
Non-ischemic chest pain	**6** (**4%**)		2 (3%)	4 (4%)	

*Dyspnea*					
7-week visit		21			NS
No dyspnea	**150** (**88%**)		59 (87%)	91 (88%)	
NYHA I-II	**9** (**5%**)		3 (5%)	6 (6%)	
NYHA III-IV	**0**		0	0	
Non-cardiac dyspnea	**12** (**7%**)		6 (9%)	6 (6%)	
12-month visit		45			NS
No dyspnea	**133** (**91%**)		54 (92%)	79 (90%)	
NYHA I-II	**5** (**3%**)		3(5%)	2(2%)	
NYHA III-IV	**1** (**1%**)		1 (2%)	0	
Non-cardiac dyspnea	**8** (**5%**)		1 (2%)	7 (8%)	

At the 7-week and 12-month visits, 88% and 91% of patients, respectively, reported no dyspnea. No differences in dyspnea patterns were observed between the sexes at either visit.

EQ-5D (3 L) data were available for 150 patients (78%) at the 7-week visit and 136 patients (71%) at the 12-month visit (Table 3). There were no significant differences in age, sex, hypertension, previous myocardial infarction, smoking status, or presenting symptoms between the 150 patients with EQ-5D data reported at the 7-week visit and the 42 patients without such data. However, diabetes was more common among those with reported EQ-5D scores (*p* = 0.04). The median EQ-5D index score was 0.86 (IQR 0.72–0.87) at the 7-week visit and 0.78 (IQR 0.69–0.87) at the 12-month visit, representing a non-significant decrease. At both visits, men reported higher EQ-5D index scores than women.

**Table 3:** Sex-differences in HRQoL at follow up.

	Total pop N = 192	Missing	Men N = 74	Women N = 118	p-Value
7-week visit					
EQ-5D index	**0.86** (**0.72**–**0.87**)	42†	0.87 (0.78–0.87)	0.78 (0.69–0.87)	=0.015
12-month visit					
EQ-5D index⁎	**0.78** (**0.69**–**0.87**)	56‡	0.87 (0.78–0.87)	0.78 (0.69–0.87)	=0.012

⁎ Comparison visit 1 and 2, *p* = 0.16.

† 14 men and 28 women.

‡ 20 men and 36 women.

**Fig. 1:**
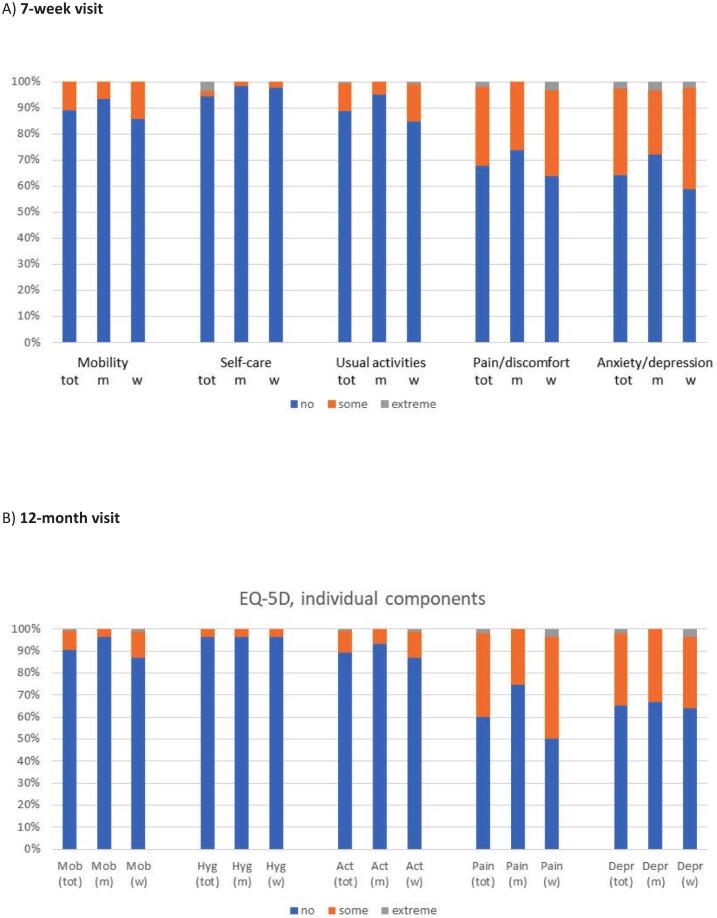
EQ-5D, individual components in the total group (tot) and in men (m) and women (w). A). EQ-5D, individual components at the 7-week visit B). EQ-5D, individual components at the 12-month visit.

The results for the individual EQ-5D components in the total study population, as well as in men and women, at the 7-week and 12-month visits are presented in Fig. 1A-B. No statistically significant differences were observed in the individual components between the 7-week visit and the 12-month visit in the overall population. Likewise, no sex-related differences were found in the individual components, except that women reported more pain at the 12-month visit (*p* = 0.01).

Supplemental Table 2 presents median EQ-5D index scores for Swedish MINOCA-BAT patients at the 7-week visit, for MI patients of similar age and LVEF >40% registered in the SWEDEHEART registry 6–10 weeks after the index MI, and for a Swedish general population of the same age [10]. Median EQ-5D index scores were similar across all three populations. Results of the individual EQ-5D components for the three groups are shown in Supplemental Fig. 1, indicating that depression/anxiety was more common among MINOCA patients than in the other groups.

Supplemental Table 3 shows the occurrence of chest pain and dyspnea in Swedish patients in the MINOCA-BAT cohort and the SWEDEHEART MI cohort. MINOCA patients were less often free of chest pain but more often free of dyspnea.

### 3.3 Effect of randomized treatment on chest pain, dyspnea and HRQoL

There were no significant differences in the occurrence of chest pain, dyspnea, or EQ-5D index scores at any visit between patients randomized to beta-blockers versus no beta-blockers, or to ACEI/ARB versus no ACEI/ARB (Supplemental Table 4).

### 3.4 Prediction of EQ-5D index score

A linear regression model predicting the EQ-5D index score at one year, based on sex, region of enrolment (Sweden/non-Sweden), age and age^2^, demonstrated limited explanatory power, with an adjusted R^2^ of 0.062. Because age showed a non-linear relationship, a squared age term (age^2^) was included in the model. Adding baseline risk factors and comorbidities (hypertension (yes/no), active smoking (yes/no), diabetes (yes/no), and previous myocardial infarction (yes/no)) increased the explanatory value only marginally, yielding an adjusted R^2^ of 0.107 (Supplemental Table 5).

## 4 Discussion

In this post hoc analysis of a contemporary cohort of patients with a working diagnosis of MINOCA and preserved LV-EF, we report three major findings: first, our enrolled cohort of patients with a working diagnosis of suspected MINOCA and preserved LV-EF was a low-risk population, with most experiencing no ongoing cardiac symptoms during follow-up and a low incidence of new cardiac events; second, at 7 weeks and 12 months after the index event, patients demonstrated HRQoL, measured by the EQ-5D index, comparable to that of age-matched individuals in the general population; third, women reported lower HRQoL compared with men after MINOCA, consistent with sex differences observed in the general population and in patients with stable CAD and stable ischemia with no obstructive coronary arteries [10], [11].

The study population exhibited fewer high-risk characteristics than previously reported in MINOCA cohorts (e.g., lower prevalence of hypertension, diabetes, and prior MI [12]. This is likely explained by the inclusion criterion of preserved LV-EF (≥40%) and the exclusion of patients with clinical signs of heart failure in the MINOCA-BAT trial, which may also account for the low incidence of clinical events during the first year compared with other studies [2], [12]. Further, clinicians at enrolling centers may have prioritized for enrolment lower risk patients in whom they had equipoise regarding the need for beta blocker and ACEI/ARB treatment.

Although MINOCA was more frequent among women, few differences in clinical characteristics and admission findings were observed between sexes, consistent with previous registry-based studies [13]. Notable differences included: 1) men more often presented without ST-T changes on admission ECG, although rates of ST-segment elevation were similar (Table 1). This may suggest a lower threshold for coronary angiography in men compared with women [13], [14]. 2) Coronary stenosis of 30–49% was more common in men, likely explaining the higher prescription rates of statins and P2Y12 inhibitors at discharge. 3) Women more frequently had mildly reduced LV-EF (40–49%), despite similar troponin levels between sexes.

### 4.1 Chest pain, dyspnea at follow-up

Only a minority reported ischemic chest pain (angina pectoris): 14% at the 7-week visit, decreasing to 8% at the 12-month visit. No significant sex-related differences were observed at either visit. However, due to the small sample size, the existence of minor differences in symptoms between the sexes cannot be ruled out. The proportion of patients reporting chest pain or angina during follow-up was 12 and 19% in a Swedish and a Polish observational study, respectively [15], [16]. In another, American study, 22% had been readmitted because of chest pain at three years, with no difference between men and women [13]. In the recently published PROMISE trial, which compared a standardized diagnostic algorithm with usual care, 80% of patients in the intervention group were free of angina pectoris at 12 months, as assessed by the Seattle Angina Questionnaire [17].

Similarly, dyspnea was reported by a minority (12% at the 7-week visit and 9% at the 12-month visit), with no sex differences. While the overall prevalence remained stable, severity appeared to increase between visits, with a greater proportion reporting more severe cardiac-related dyspnea at the 12-month visit. To our knowledge, no previous study has reported the prevalence of dyspnea at follow-up after MINOCA.

### 4.2 Health related quality of life at follow-up

The EQ-5D index score decreased numerically but not significantly between the 7-week and 12-month visits. These findings are consistent with a systematic review of MINOCA and Takotsubo syndrome [6].

EQ-5D index scores in Swedish MINOCA-BAT patients were similar to those of MI-CAD patients of the same age with preserved LV-EF in the SWEDEHEART registry, and surprisingly, comparable to a small sample from the general population [10]. Anxiety and depression were most frequently reported in the MINOCA group and least in the general population. Furthermore, EQ-5D index scores during follow-up were comparable to those reported in a study of 9474 MI patients admitted to 77 hospitals in England [18]. However, another Swedish study found MINOCA patients scored lower than population-based controls in role functioning (physical, social, emotional) at 12 months using RAND-36 [19]. Differences between studies may reflect the use of different HRQoL instruments and the lower baseline risk in MINOCA-BAT. However, the comparisons with the data from SWEDEHEART and general population should be regarded as hypothesis generating, especially as the general population sample is small and not contemporary.

Men reported higher EQ-5D index scores than women at both visits. In individual EQ-5D dimensions, men reported fewer problems across all domains, with statistical significance only in pain at the 12-month visit. This aligns with general population data, where similar sex differences exist but are less influential than age and other socio-demographic factors [10]. Studies of MI-CAD have similarly shown lower HRQoL in women compared with men at baseline and during follow-up [18], [20], [21].

Although the study cohort was too small to assert any conclusions regarding randomized study medications, there were no differences in EQ-5D index scores between patients randomized to the active treatments and corresponding controls. The results are nevertheless consistent with observations in MI-CAD patients with preserved LV-EF, where HRQoL did not differ by beta-blocker therapy [22].

Age, sex, and traditional cardiac risk factors had limited predictive value for EQ-5D index scores in this low-risk MINOCA cohort. Other factors, such as history of mental illness, comorbidities affecting daily activities, socioeconomic status, and social support [10], [23], likely play a greater role in perceived quality of life also in MINOCA patients.

### 4.3 Limitations

The present study has several limitations. First, because the MINOCA-BAT trial was prematurely terminated, the sample size is limited. Nevertheless, the number of patients included is comparable to most previous studies on HRQoL in MINOCA [6]. Second, the trial was designed before publication of the first clinical guideline clearly recommending cardiac magnetic resonance or other advance coronary imaging or testing as part of the routine diagnostic work-up [24]. Consequently, magnetic resonance imaging, optical coherence tomography, or vasospasm testing were not routinely performed or recorded. It is therefore possible that some patients with myocarditis or Takotsubo syndrome—conditions not evident at the time of randomization—were misclassified as having MINOCA. The findings should thus be interpreted as applying to patients with a *working* diagnosis of suspected MINOCA. Third, EQ-5D data were missing for a number of patients which introduces the possibility of responder bias and therefore the comparisons with the general population and the SWEDEHEART cohort should be interpreted cautiously; however, clinical characteristics differed only minimally between those with and without reported EQ-5D data, and the proportions of missing EQ-5D data were similar in men and women (visit 1: 19 vs. 24%, *p* = 0.43; visit 2: 27 vs. 31%, *p* = 0.61). Fourth, we used the EQ-5D (3 L) questionnaire, which is widely employed and well validated [10]. However, EQ-5D has been criticized for its limited sensitivity in detecting more subtle changes in HRQoL [25], a limitation that may be especially relevant when assessing the impact of medication on HRQoL. Fifth, because no adjustment of *p*-values for multiple comparisons was performed, there is a risk of Type I errors. Marginally significant differences should therefore be interpreted with caution and require confirmation in future studies. Sixth, because of the small number of patients and clinical events, we were unable to examine the association between HRQoL and the risk of mortality or other cardiac events after MINOCA. Such an association between HRQoL and mortality risk has previously been demonstrated in a general MI population [18]. Seventh, the proportions of patients randomized to beta-blockers and/or ACEI/ARB who had discontinued treatment at one year were relatively high, but comparable to what has previously been reported in a large MINOCA population [26].

### 4.4 Clinical implications

The subgroup of patients with a working diagnosis of MINOCA and preserved LV-EF appears to have a favorable prognosis, with only minor differences between men and women in the risk of quality of life and residual symptoms. These findings are relevant for informing the management of this low-risk population but should not be extrapolated to patients with higher-risk characteristics, such as impaired left-ventricular function.

## CRediT authorship contribution statement

**Bertil Lindahl:** Resources, Project administration, Methodology, Funding acquisition, Formal analysis, Data curation, Conceptualization, Writing – review & editing. **Anna Nordenskjöld:** Project administration, Data curation, Conceptualization, Writing – review & editing. **Sivabaskari Pasupathy:** Project administration, Data curation, Writing – review & editing. **Rosanna Tavella:** Project administration, Data curation, Writing – review & editing. **Stefan Agewall:** Supervision, Writing – review & editing. **Dan Atar:** Supervision, Project administration, Writing – review & editing. **Tomasz Baron:** Conceptualization, Writing – review & editing. **David Erlinge:** Supervision, Writing – review & editing. **Chris P. Gale:** Supervision, Writing – review & editing. **Tomas Jernberg:** Supervision, Writing – review & editing. **Felicia Håkansson:** Supervision, Writing – review & editing. **Pelle Johansson:** Methodology, Writing – review & editing. **Javier López Pais:** Supervision, Writing – review & editing. **Annica Ravn-Fischer:** Supervision, Writing – review & editing. **Harmony Reynolds:** Supervision, Writing – review & editing. **Jithendra Somaratne:** Project administration, Data curation, Writing – review & editing. **John Beltrame:** Supervision, Project administration, Methodology, Funding acquisition, Conceptualization, Writing – review & editing. **Olle Bergström:** Writing – review & editing.

## Ethical statement

The MINOCA-BAT trial was registered in ClinicalTrials.gov (NCT03686696) and approved by the appropriate Ethical Review Boards in each participating country, in accordance with the Declaration of Helsinki principles.

## Funding

MINOCA-BAT was funded by the 10.13039/501100004359Swedish Research Council (2017-00478) and the Hospital Research Foundation 2017 Translational Research Grant Funding, Adelaide, Australia.

## Declaration of competing interest

Bertil Lindahl has received research grants from the Swedish Research Council and the Swedish Heart and Lung Foundation.

Dan Atar has received speaker fees from Abbott, Actelion, Amgen, Amarin, AstraZeneca, Bayer, BMS, Boehringer-Ingelheim, GSK, Idorsia, MSD, Novartis, NovoNordisk, Pfizer, Pharmacosmos, Philips, Roche-Diagnostics, Sanofi, Takeda, Viatris, Vifor; and grant support (to the Institution) from BMS/Pfizer, Medtronic, Bayer, Roche-Diagnostics.

David Erlinge has received honorarium for advisory board/speaker fees from AstraZeneca, Sanofi, Amgen, NovoNordisk, Bayer, InfraredX/Nipro and Kaminari Medical.

Chris Gale has received honorarium from Amgen, AstraZeneca, Bayer, BMS, Boehringer-Ingelheim, GSK, MSD, Novartis and grant support (to the Institution) from Abbott, BMS/Pfizer, Daiichi-Sankyo all outside of the submitted work.

Jithendra Somaratne has received a supplementary grant from the Green Lane Research and Educational FundJohn Beltrame

Anna Nordenskjöld, Sivabaskari Pasupathy, Rosanna Tavella, Stefan Agewall, Tomasz Baron, Olle Bergström, Tomas Jernberg, Felicia Håkansson, Pelle Johansson, Javier López Pais, Annica Ravn-Fischer and Harmony Reynolds report no conflict of interest in relation to the present study.

## Appendix A Supplementary data

Supplemental Fig. 1 Comparison of individual components of EQ-5D between different populations, MINOCA-BAT, MI-patients aged 55–64 years and with LVEF >40% in SWEDEHEART and a general Swedish population aged 55–64 years.
