# Supplementary material for: Health related quality of life and cardiac symptoms following admission for suspected MINOCA: A post-hoc analysis of the MINOCA-BAT randomized clinical trial
Source: Am Heart J Plus. 2026 Aug 5;70:100856. doi: 10.1016/j.ahjo.2026.100856 (PMC13475666; doi:10.1016/j.ahjo.2026.100856)

Supplemental figure 1

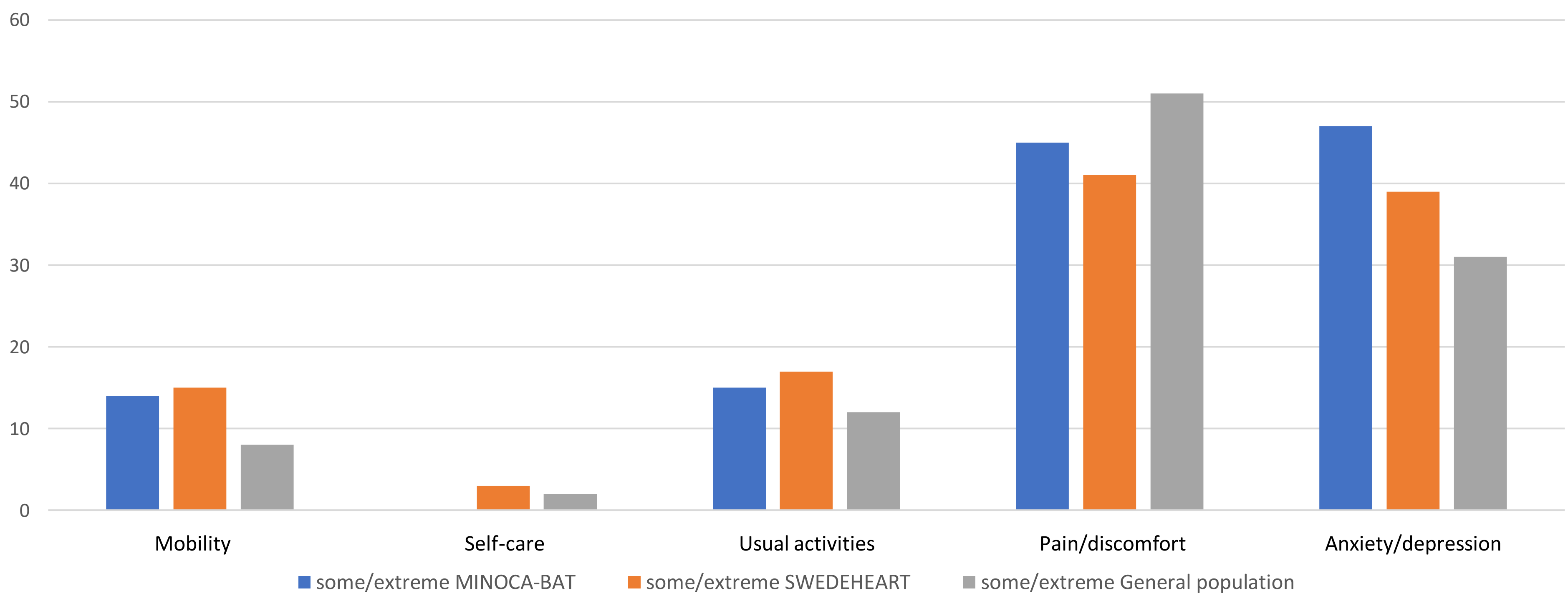

Supplement: Supplementary file 1 [file mmc1.pdf]
