# Supplementary material for: Health related quality of life and cardiac symptoms following admission for suspected MINOCA: A post-hoc analysis of the MINOCA-BAT randomized clinical trial
Source: Am Heart J Plus. 2026 Aug 5;70:100856. doi: 10.1016/j.ahjo.2026.100856 (PMC13475666; doi:10.1016/j.ahjo.2026.100856)
Supplement: Supplementary file 2 [file mmc2.docx]

## Supplemental tables

## Supplemental table 1. Medications at discharge

|  | **Total pop**  **N=192** | **missing** | **Men**  N=74 | **Women**  N=118 | **p-value** |
| --- | --- | --- | --- | --- | --- |
| Ca-inhibitor | **30 (16%)** | 1 | 10 (14%) | 20 (17%) | NS |
| Long-acting  Nitrate | **3 (2%)** | 1 | 0 | 3 (2%) | NS |
| Diuretics | **7 (4%)** | 1 | 4 (5%) | 3 (2%) | NS |
| MRA | **2 (1%)** | 1 | 0 | 2 (2%) | NS |
| Statins | **151 (79%)** | 1 | 65 (88%) | 86 (73%) | P=0.008 |
| Ezetimibe | **8 (4%)** | 1 | 4 (5%) | 4 (3%) | NS |
| Diabetes  treatment | **8 (4%)** | 1 | 4 (6%) | 4 (3%) | NS |
| Aspirin | **169 (88%)** | 1 | 67 (91%) | 102 (86%) | NS |
| P2Y12-inh. | **89 (47%)** | 1 | 45 (62%) | 44 (37%) | =0.001 |
| Anticoagulant treatment | **7 (4%)** | 1 | 2 (3%) | 5 (4%) | NS |

## Supplemental table 2

EQ-5D

|  | **MINOCA-BAT,**  **Sweden, 7-week visit**  **N=82** | **SWEDEHEART,**  **6-10 w**  **N=21,130** | **Swedish general population**  **N=70** |
| --- | --- | --- | --- |
| EQ-5D index | 0.78 (0.69-0.87) | 0.78 (0.69-0.87) | 0.78 (0.69-1.00) |

SWEDEHEART. Measurements 6-10 weeks after a MI (all types), patients aged 55-64 and with LVEF >40%,

Swedish normal population age 55-65 years (Björk 1999)

## Supplemental table 3

|  | **MINOCA-BAT,**  **Sweden, 7‑week visit**  **N=98** | **SWEDEHEART,**  **6-10 w**  **N=21,645** |
| --- | --- | --- |
| ***Chest pain*** | | |
| No chest pain | 80 (81%) | 18352 (85%) |
| CCS I-II | 12 (12%) | 1960 (8%) |
| CCS III-IV | 0 | 170 (0.7%) |
| Non-ischemic  chest pain | 7 (7%) | 1163 (5%) |
| ***Dyspnea*** | | |
| No dyspnoea | 89 (91%) | 17517 (81%) |
| NYHA I-II | 3 (3%) | 3118 (14%) |
| NYHA III-IV | 0 | 298 (1%) |
| Non-cardiac  dyspnea | 6 (6%) | 678 (3%) |

## Supplemental table 4. Randomized treatment and QoL, chest pain and dyspnea (total study population)

|  | Randomized to beta-blocker | Randomized to no beta-blocker | *p-value* | Randomized to ACEI/ARB | Randomized to no ACEI/ARB | *p-value* |
| --- | --- | --- | --- | --- | --- | --- |
| **EQ-5D index**  **7-week visit**  **12-month visit** | *0.87 (0.78-0.87)*  *0.78 (0.73-0.87)* | *0.78 (0.69-0.87)*  *0.78 (0.69-0.87)* | *NS*  *NS* | *0.87 (0.70-0.87)*  *0.78 (0.69-0.87)* | *0.78 (0.75-0.87)*  *0.78 (0.70-0.87)* | *NS*  *NS* |
| **Chest pain**†  **7-week visit**  **12-month visit** | 15%  14% | 24%  14% | NS  NS | 23%  13 % | 17%  15% | NS  NS |
| **Dyspnea**†  **7-week visit**  **12-month visit** | 10%  14 % | 14%  5% | NS  0.09 | 9%  8 % | 16%  11% | NS  NS |

† Any chest pain and dyspnea, respectively.

## Supplemental table 5 - Linear regression models for prediction of EQ-5D index score at one year.

|  | Beta | Standard error | p-value |  | Beta | Standard error | p-value |
| --- | --- | --- | --- | --- | --- | --- | --- |
| Constant | 0.562 | 0.274 | 0.043 |  | ,555 | ,270 | ,042 |
| sex | -0.047 | 0.021 | 0.031 |  | -,048 | ,021 | ,027 |
| age | 0.012 | 0.009 | 0.204 |  | ,013 | ,009 | ,150 |
| age^2^ | 0.000 | 0.000 | 0.163 |  | ,000 | ,000 | ,111 |
| region | -0.024 | 0.022 | 0.275 |  | -,045 | ,023 | ,056 |
| diabetes |  |  |  |  | -,019 | ,012 | ,120 |
| hypertension |  |  |  |  | ,000 | ,023 | ,989 |
| prev. MI |  |  |  |  | ,067 | ,046 | ,146 |
| smoking |  |  |  |  | -,058 | ,026 | ,029 |
